# A Cyclopædia of Drug Pathogenesy*

**Published:** 1887-08

**Authors:** George A. Taber

**Affiliations:** Richmond, Va.


### A CYCLOPÆDIA OF DRUG PATHOGENESY.*

"*Heu prisca fides.*"

George A. Taber, M. D., Richmond, Va.

In the fifth American edition of the *Organon*, page 120, § 106, we read : " The entire range of disease-producing power of each drug must be known, that is, all morbid symptoms and changes of the state of health which each drug is capable of producing by itself in healthy persons should have been observed in its fullest extent, before we may hope to find and to select from among the medicines thus investigated the truly homœopathic remedies for most natural diseases."

The compilation of this *Cyclopædia of Drug Pathogenesy* is taking place under instructions adopted jointly by the American Institute of Homœopathy and the British Homœopathic Society. The second ruling of these instructions provides that the compilers "give a narrative of all provings, stating the symptoms in the order of their occurrence, with such condensation as completeness allows." I can see no conflict in the requirements of the Master and the demands of his representatives, for the paragraph from the *Organon* above cited and the second ruling of the Societies named are in strict accord. Does this *Cyclopædia of Drug Pathogenesy* meet these requirements? The reader, with the foregoing demands constantly in mind as a guide, may be an impartial judge by noting the following comparison, from the original day-book of G. A. Taber, in his third

---

* Edited by Richard Hughes, M. D., and J. P. Dake, M. D.

proving of Picric acid, and the purported record of the same in the *Cyclopœdia of Drug Pathogenesy.*

| Picric Acid. | Picric Acid. |
|---|---|
| Third proving by G. A. Taber, from his original *day-book.** | Third proving by G. A. Taber as recorded in *Cyclopœdia of Drug Pathogenesy.* |
| For two days he took ten grain doses of the 3x and 2x trits. On the third day took two ten grain doses of the 2x, and four doses, same size, of the 1x trits., from the fourth day to the end increasing doses of the 1x trit. | For third day took 3x and 2x trits. From fourth to tenth day increasing doses of 1x. |
| SECOND DAY. | SECOND DAY. |
| A shooting pain from right eyeball to right side of occiput, lasting but a few seconds (ten minutes after taking fourth dose for the day). | |
| THIRD DAY. | THIRD DAY. |
| Dull pain in right lower occipital region, beginning at quarter after eleven A. M. and lasting till half-past twelve P. M. The pain came on gradually and as gradually subsided. | On third day occipital pain recurred (for one and three-fourth hours), and again on fourth day (for one hour). |
| FOURTH DAY. | FOURTH DAY. |
| A dull pain in cardiac region of stomach while eating breakfast at seven A. M.<br>Pain in right lower occipital region at eight A. M., lasting about an hour, and at the same time a sensation as of a band pressing † along the right parietal eminence. | On this day dull pain at cardiac orifice of stomach while breakfasting. |
| FIFTH DAY. | FIFTH DAY. |
| At quarter after nine A. M. dull pain in right lower occipital region, which comes and goes at short intervals.<br>Four or five times during the afternoon a dull pain would come in calf of right leg, occupying a space about the size of a silver half dollar. It would last but a few seconds, then an interval of relief followed by a return of the pain. | On fifth occipital pain came and went all day; in morning more frequent micturition than usual. At one P. M. prostrate feeling after short walk. |

* The wording of symptoms will be as in Allen unless otherwise stated.
† In Allen this is wrongly printed passing.

| | |
|---|---|
| FIFTH DAY—CONTINUED. | FIFTH DAY—CONTINUED. |
| At one P. M. a prostrate feeling came over me while walking a short distance, which was quite unnatural to me.<br>Frequent micturition this afternoon.* | |
| SIXTH DAY. | SIXTH DAY. |
| At quarter of eight A. M. pain in calf of right leg at short intervals; the spot feels a little sore on hard pressure. During the morning more frequent micturition than usual.<br>Prostration on slight exertion. | On sixth day same symptoms without headache; also (and on fifth day as well) frequent dull pain on small spot in right calf, which at length became tender. |
| SEVENTH DAY. | SEVENTH DAY. |
| Eyes to-day markedly yellow, being stained by the acid. | On seventh day yellowness of eyes. |
| EIGHTH DAY. | EIGHTH DAY. |
| Slight nausea for an hour or two after taking the drug.<br>Prostration of mind after writing for a time.<br>At one P. M. pain in lower right occipital region, coming on at intervals since eleven A. M. | On the eighth day prostration of mind after writing a while; occipital pain; slight nausea for one or two hours after taking drug. |
| NINTH DAY. | NINTH AND TENTH DAYS. |
| A dull pain began in right lower occipital region at eight A. M. and continued two hours. | On ninth and tenth days occipital pains. |
| TENTH DAY. | |
| No symptoms recorded on this day, and not again until the eighteenth day. | |
| EIGHTEENTH DAY. | EIGHTEENTH DAY. |
| † Dull pressing pain in right suboccipital region, beginning about four P. M. and lasting until ten P. M. | |

In commenting upon the foregoing comparison let us begin at the record of doses, an unimportant item perhaps. However, the *Cyclopædia* is in error even in this matter.

* Allen wrongly gives this as occurring in the morning, corresponding with a similar symptom on the sixth day.

† This is given in Allen as a part of symptom 94.

On the second day may be noted on the part of the *Cyclopædia* "condensation" very much at the expense of "completeness." On the third day in the *Cyclopædia* record we have in the first place an error of thirty minutes, and, second, a symptom recorded that occurred on the fourth day; this much at the expense of "stating the symptoms in the order of their occurrence."

As the record of the *Cyclopædia* runs, on the fourth day please note how much more definite and precise the cardiac pain is located than the prover dared to express; and also on this day no mention is made of the peculiar occipital pain the prover records; "condensing" again at the expense of "completeness."

As we enter upon the *Cyclopædic* record for the fifth day, the reader, as the writer, will soon find "confusion worse confounded." Shades of Tom Paine, of Voltaire, and of Bob Ingersoll when he dies, what transcribing! a symptom that the prover records at fifteen minutes past nine in the morning, the *Cyclopædia* makes occur all day, and the frequent afternoon micturition of the prover's record is a *Cyclopædic* morning symptom. The prover's further record of this day says—a prostrate feeling came over me *while** walking: "stating the symptoms i n the order of their occurrence," and "condensation as completeness allows," in their violation offer no comparison on this *Cyclopædic* day to its record of "prostrate feeling *after** short walk."

On the sixth and eighth days the *Cyclopædia* again violates the record in the order of the occurrence of symptoms; and on the tenth day gives occipital pains, while the original day-book records no symptoms whatever on this day. Please notice, we omitted to speak of the seventh and ninth days; they are so nearly correct we wished to give the *Cyclopædia* credit for giving two days' record from a total of ten almost right.

Fellow class-mates of '77, from the University of Michigan, Homœopathic Department, is this the *Cyclopædia of Drug Pathogenesy* our teacher in materia medica longed for? Do we realize in this his description of what we, as students of materia medica, needed? Do we find in it "a narative of all provings, stating the symptoms in the order of their occurrence, with such condensation as completeness allows"?

> Garmon, they have changed your J. to S.,
> And Adams' E. to G.,
> And all our symptoms are in a mess
> From A way down to Z.

---

* The italics ours.

